# Advancements in Heavy Metal Stabilization: A Comparative Study on Zinc Immobilization in Glass-Portland Cement Binders

**DOI:** 10.3390/ma17122867

**Published:** 2024-06-12

**Authors:** Abdelhadi Bouchikhi, Amine el Mahdi Safhi, Walid Maherzi, Yannick Mamindy-Pajany, Wolfgang Kunther, Mahfoud Benzerzour, Nor-Edine Abriak

**Affiliations:** 1 Laboratoire des Infrastructures Intelligentes et des Technologies de l’Environnement Connectés (LabI2TEC), Institut Supérieur du Bâtiment et des Travaux Publics, F-13009 Marseille, France; 2 Department of Building, Civil, and Environmental Engineering, Concordia University, Montreal, QC H3G 1M8, Canada; amineelmahdi.safhi@concordia.ca; 3 Institut Mines-Télécom Lille Douai, Université de Lille, ULR 4515—LGCgE, F-59000 Douai, France; walid.maherzi@imt-nord-europe.fr (W.M.); yannick.mamindy@imt-lille-douai.fr (Y.M.-P.); mahfoud.benzerzour@imt-nord-europe.fr (M.B.); nor-edine.abriak@imt-nord-europe.fr (N.-E.A.); 4 Materials and Durability, Department of Environmental and Resource Engineering, Technical University of Denmark, 2800 Kongens Lyngby, Denmark; wolku@dtu.dk

**Keywords:** zinc stabilization, ground glass, supplementary cementitious materials, sorption isotherms

## Abstract

Recent literature has exhibited a growing interest in the utilization of ground glass powder (GP) as a supplementary cementitious material (SCM). Yet, the application of SCMs in stabilizing heavy metallic and metalloid elements remains underexplored. This research zeroes in on zinc stabilization using a binder amalgam of GP and ordinary Portland cement (OPC). This study juxtaposes the stability of zinc in a recomposed binder consisting of 30% GP and 70% OPC (denoted as 30GP-M) against a reference binder of 100% CEM I 52.5 N (labeled reference mortar, RM) across curing intervals of 1, 28, and 90 days. Remarkably, the findings indicate a heightened kinetic immobilization of Zn at 90 days in the presence of GP—surging up to 40% in contrast to RM. Advanced microstructural analyses delineate the stabilization locales for Zn, including on the periphery of hydrated C_3_S particles (Zn–C_3_S), within GP-reactive sites (Si*–O–Zn), and amid C–S–H gel structures, i.e., (C/Zn)–S–H. A matrix with 30% GP bolsters the hydration process of C_3_S vis-à-vis the RM matrix. Probing deeper, the microstructural characterization underscores GP’s prowess in Zn immobilization, particularly at the interaction zone with the paste. In the Zn milieu, it was discerning a transmutation—some products born from the GP–Portlandite reaction morph into GP–calcium–zincate.

## 1. Introduction

Intensive research previously conducted on recycled ground glass powder (GP) as supplementary cementitious materials (SCMs) [1,2,3,4] has led to the normalization of using these materials as additives. ASTM C1866–2020 Standard has established a specification for ground-glass pozzolan used in concrete [5]. Indeed, their use has proven to be beneficial in stabilizing the structure of concrete and decreasing its carbonation and chloride diffusion [6,7]. Ground-glass pozzolan has been used both in conventional cementitious matrixes and as a source of silicate in alkali-activated binders [8,9,10,11]. Nevertheless, the effect of GP on the stabilization of pollutants, such as metallic and metalloid trace elements (MMTEs), and on organic pollutants has not been reported to date. The reactivity and constituted phases of hydrated binders are among the keys to understanding the modes and locations of pollutants fixed on a solid support. Most aluminosilicate compounds are characterized by regular micropores in the form of connected channels, where pollutants preferentially accumulate. This structural state also provides a strong ionic exchange capacity between the alkalis present in zeolites (K^+^, Na^+^, and Ca^2+^) and the metallic pollutants present in water (Ni^2+^, Zn^2+^, Cu^2+^, As^5+^, U^6+^, Cr^3+^, Pb^2+^, and Cs^+^) [12,13,14,15]. In this type of fixing medium, cation exchange leads to extremely stable and sometimes irreversible bonds. However, these cationic exchanges are influenced by certain parameters in the matrix, particularly the pH, nature of the binder (Portland, geopolymer, or polymer), cure time, and degree of hydration [16,17,18].

Other compounds, such as sepiolite, geopolymeric compounds, and polymers, can also stabilize metal pollutants [19,20,21,22]. Cement hydration phases are also known for their ability to stabilize various types of pollutants. Moudilou’s work on the stabilization of MMTE using Portland CEM I 52.5 N enabled the phases involved in the binding of transition metals to be determined [23]. It appears that elements such as Ni^2+^, Zn^2+^, Cu^2+^, and Pb^2+^ are extremely stable in C–S–H gel (calcium silicate hydrate), while Cr^3+^ and U^6+^ are extremely stable in ettringites. Other elements are rather soluble in the interstitial water and hydroxide phases. Cu^2+^ and Pb^2+^ are particularly well adsorbed on C–S–H. Likewise, they are involved in the crystallographic structure of C–S–H, while Cd^2+^, Ni^2+^, and Zn^2+^ remain attached to the hydroxide phases and in the inter-granular porosities [24]. There are three mechanisms for immobilizing MMTE, including sorption, chemical incorporation, or encapsulation [19,25,26].

These attachment modes of the elements to the hydrated cementitious phases involve modifications to the crystal structure of the formed phases, heat of hydration, setting times, and durability of the matrix, including the release of the heavy metals. The presence of water plays an important role in the transport, ionization, and hydration of the substrate surface and pollutants. This allows for other types of interaction with the solid, such as adsorption, precipitation, substitutions, and inclusion. Pollutants can interact with the cement phases in several ways, including adsorption as an outer sphere complex, formation of an internal sphere complex, diffusion in the crystal lattice and isomorphic substitution, rapid lateral diffusion and formation of a surface polymer, adsorption on a mineral growth front, and surface polymer formation and incorporation into the host matrix after crystal growth. The adsorbed ion can optionally be released back into the solution by, e.g., following surface redox reactions or dynamic equilibrium. The solvency of water also influences this balance of the presence of Zn in the different phases [27].

In France, the concentrations of MMTE in wastes (e.g., sediments and industrial scraps) indicate that zinc is present at rates exceeding 5–10 times the concentrations of other MMTEs and heavy metals. To address this issue, several studies have been conducted to examine the influence of Zn in cementitious binders [28]. This study aims to analyze the effect of GP as an SCM on the binding behavior of Zn^2+^ in different hydration phases. To do this, we evaluated a matrix with a 70:30 ratio of cement to GP and a reference matrix (100% cement). Furthermore, mechanical, absorption, isothermal, and microstructural studies were conducted as a function of time. The objective of using GP is to study its influence on both the pozzolanic activity (by monitoring the compressive strength compared to a 100% cement matrix) and the stabilization of active Zn (reactivity within the matrix, i.e., pollutant immobilization).

## 2. Materials and Methods

### 2.1. Materials

The particle size distribution (PSD) analyses were conducted using the Mastersize 2000 laser (Malvern Panalytical, Malvern, UK) in dry mode. This instrument facilitates the determination of PSD spanning from 0.04 μm to 2000 μm. The test adheres to the ISO 13320 standard [29]. The cement used in this study was CEM I 52.5 N, containing 95% clinkers and a 5% calcium sulfate additive. The GP was crushed finely on a laboratory scale (d_90_ < 15 µm) before being washed with demineralized water. To ensure high solubility of the contaminant in water, an ultra-pure zinc sulfate hexahydrate (Zn(N_2_O_6_)_2_,6H_2_O) was used. To ensure a homogeneous presence of Zn in the paste, the pollutant was dissolved beforehand in the mixing water. Different percentages by weight of the binder (¼, ½, 1, 2, and 3%) of Zn(SO_4_)_2_ were added. Normalized sand, in accordance with the standard CEN 196-1 ISO [30], was used to produce 40 × 40 × 160 mm^3^ mortars.

### 2.2. Methods

**Figure 1:**
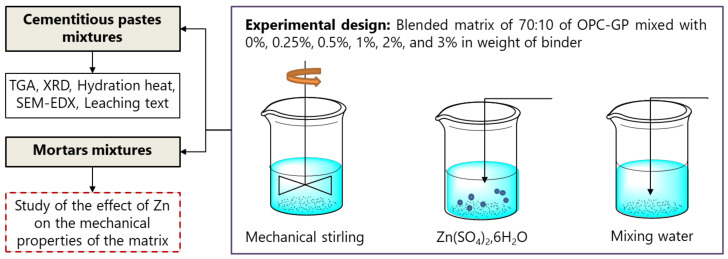
Experimental design that was adopted in this study.

The compressive strength was tested at different ages of curing (1, 7, 28, and 90 days) on three cubes of 40 mm^3^, prepared according to EN 196-1 standard [30] and using an INSTRON press (Norwood, MA, USA). The stabilization of Zn was monitored by measuring the leached Zn in demineralized water at 20 ± 2 °C using a ROTAX instrument (VELP Scientifica Srl, Usmate Velate, Italy) programmed to run for 1 turn per minute (a leaching test on a 1:10 ratio of solid to liquid) using inductively coupled plasma mass spectrometry (ICP-MS) analysis employing an Agilent Technologies SPS4 Autosampler. Thermogravimetric analysis (TG) was performed with a QMS 403D-NETZSCH machine (NLIR ApS, Farum, Denmark) using argon as the atmosphere at a flow rate of 20 mL/min. X-ray diffraction analysis (XRD) was performed using a D8 Focus diffractometer from Bruker (Billerica, MA, USA) that was equipped with a cobalt anode (λKα1 = 1.74 Ǻ) and Lynx Eye detector. Scanning Electron Microscopy (SEM) analysis was conducted using a Hitachi S-4300SE/N (Hitachi, Chiyoda-ku, Tokyo). The heat of hydration evolution was measured on pastes. Figure 1 illustrates the experimental design adopted in this study.

### 2.3. Data Analysis

The Zn distribution between the liquid phase and the binder sorbent (adsorption on surfaces) was calculated using Equation (1). The first-order Lagergren equation, the second-order kinetics model, and the Bingham equations are presented in linear form in Equations (2) (second order) and (3) (pseudo-second order). Here, *qt* is the sorbed concentration in μg/mg at time *t* (h), *qm* is the pseudo-equilibrium sorbed concentration (μg/mg), and *k*_1_ (h^−1^), *k*_2_ (g·g^−1^ h^−1^), and *k_b_* (g·g^−1^ h^−1^) are the sorption rate constants. In Equation (1), *K_d_* is the solid–liquid distribution coefficient (L/kg), *q_s_* is the sorbed concentration (mg/kg), and *q_e_* is the equilibrium concentration (mg/L).

*LogK_d_* = *log(q_s_*·*q_e_*) (1)

(2) $$\log\left(qt\right)=log\left(k_{d}\right)+\frac{1}{n}log(t)$$

(3) $$\frac{t}{qt}=\frac{1}{k_{2}qm^{2}}+\frac{1}{qm}t$$

## 3. Results and Discussion

### 3.1. Physicochemical Properties

**Figure 2:**
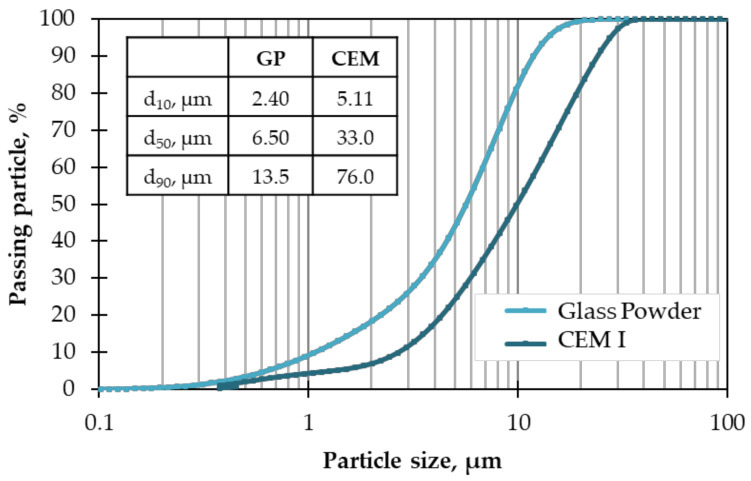
PSD of GP and CEM.

The physicochemical properties of GP and CEM are presented in Table 1. The GP is mostly composed of SiO_2_ (71 wt.%), 11 wt.% Na_2_O, and 13 wt.% CaO, with low contents of MgO, Fe_2_O_3_, SO_3_, and K_2_O. The cement is principally composed of CaO (61 wt.%), SiO_2_ (18 wt.%), Al_2_O_3_ (4.4 wt.%), SO_3_ (3.7 wt.%), and Fe_2_O_3_ (3.0 wt.%). The cement phases included 59% of C_3_S, 13% of C_2_S, 8.8% of C_3_A, and 10% of C_4_AF using Bogue formulas. The PSD of those powders is presented in Figure 2.

### 3.2. Compressive Strength Evolution

**Figure 3:**
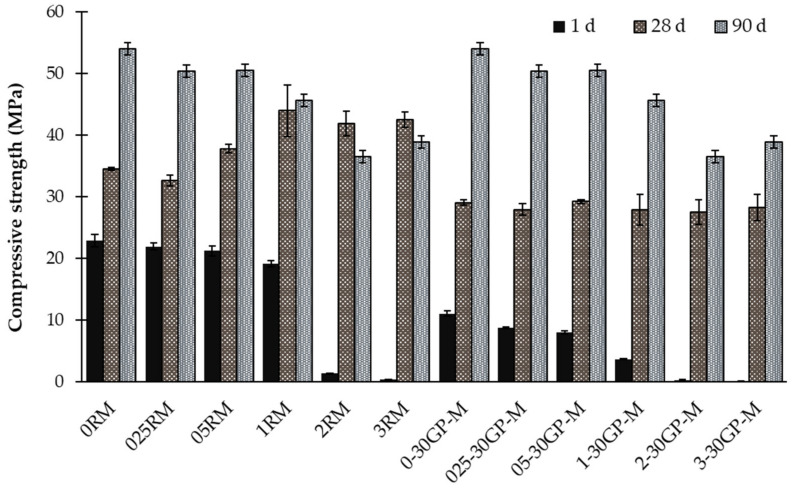
Compressive strength as a function of Zn-to-cement weight.

The compressive strengths obtained between 1 and 90 days indicate changes as a function of time and dosage of Zn (Figure 3). After 1 day of curing, the samples containing 2% and 3% of Zn showed large decreases in strength as low as −23 MPa compared to the reference matrix (0RM) (90% compared to 0% of Zn). In these samples, the slow hydration leads to reduced zinc stabilization in the mortars. These results are correlated with an extremely low heat of hydration and are reflected in the setting delay by more than 40 h compared to the 3 h for 0RM. A progressive decrease was observed in UCS of approximately −7 MPa between the 0.25% and 1% dosages. For comparison, 1% of Zn in a hydraulic cementitious mix affects the compressive strength of the hydraulic matrix equivalently to a 25% substitution of fly ash or glass aggregates [1,35]. After 90 days of curing, while all the samples showed improvements in compressive and flexural strengths, the 30% GP-mortars were slightly better compared with the reference matrix series.

### 3.3. Fixation Isotherms

**Figure 4:**
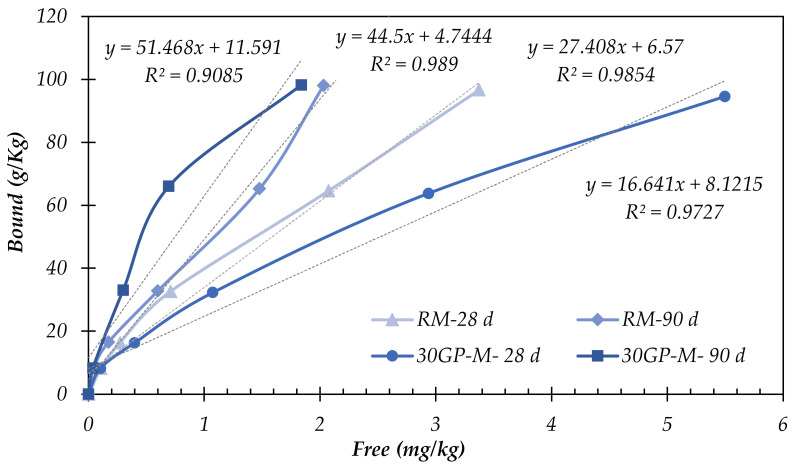
Fixed Zn in the function leached Zn in the samples.

The leaching test results at 28 and 90 days (Figure 4) represent the importance of curing time to the fixation of Zn, which is much more important at later ages. At 20 days of curing at 20 °C and RH > 90%, Zn is more stabilized in the reference mortar (RM) than in the composed-binder mortar 30GP-M. However, at 90 days of curing, Zn was more stabilized in the composed-binder matrix. This improvement in the fixation of the blended matrix is linked to the reactivity of the GP in the cement paste after 90 days. At 28 days of curing, the RM produced more C–S–H gel compared to the 30GP-M, resulting in more Zn incorporated or adsorbed in the C–S–H gel silicate sheets in the RM. The presence of Zn in a bivalent cationic state, Zn(II), allows an immediate reaction and integrates the C–S–H gel structures. This strong reactivity is mainly linked to the solubility of Zn(SO_4_)_2_ (96.5 g/100 g of water at 20 °C) and to a very basic pH in ordinary Portland cement. Through long curing times (explained by the slow kinetics of GP), GP reacts as a pozzolan and interacts with the Portlandite, as well as with the different forms of Zn, such as Zn(OH)_2_ and ZnCa(OH)_2_.

The stabilization of Zn is linked to the duration of the curing time. To establish this principle, a fixation study was conducted. The graphical presentation of *log*(*Qt*) as a function of *log*(*t*) was calculated according to the secondary derivative of the Bangham equation as follows:

(4) $$\log Qt=\log\left(kb\right)+\frac{1}{n}*\log\left(t\right)$$

(5) $$\log Qt=\log\left(kd\right)+\frac{1}{n}*\log\left(t\right)$$

**Figure 5:**
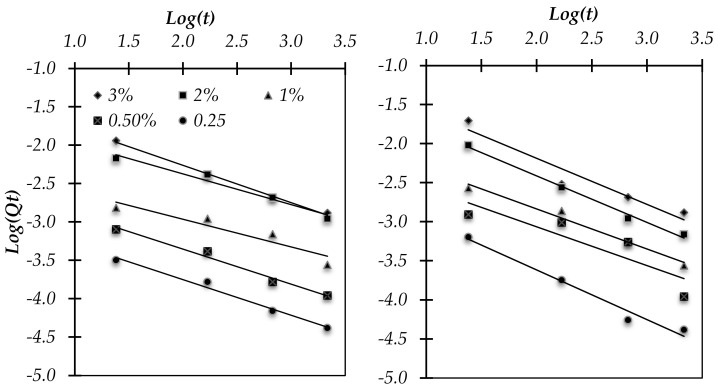
Bangham fits (solid lines) for adsorption of Zn as a function of time for each concentration of Zn. (**left**) in the MR hydrated binder and (**right**) in the 30GP-M hydrated binder.

The linear relationship between (*q_t_*) and log(t), which allows for the determination of the constant solid–liquid distribution coefficient (*k_d_*), indicates the capacity of the binder to sorb Zn. Table 2 and Figure 5 present the linear equations, *k_b_* coefficients, and R^2^ for Equation (4) above. The low concentration of Zn in the medium led to an increase in the binding capacity of the binders hydrated with 0.25 and 0.5% Zn. High concentrations of Zn (2–3%), which delay the setting and hydration of the binders, hinder its stabilization.

The reactivity of GP in the cementitious medium is linked to the interface between the GPs and contaminated paste by Zn^2+^. The nucleation site density at the surface determines the glass’s capacity for stabilization. For this, the *k_d_* of the glass is linked to its specific surface area rather than the quantity or mass of substitution.

**Figure 6:**
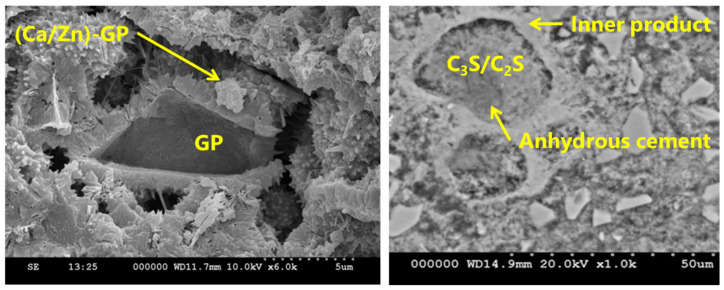
SEM observations: (**right**) anhydrous cement, (**left**) Zn–glass reaction at 90 days.

The precipitation of an amorphous layer of Zn(OH)_2_ and the hydroxide crystalline calcium zinc around the cement particles suggest very little pozzolanic activity over time. This explains the loss of mechanical properties observed with the increase in Zn dosage (Figure 6). The compression strength obtained at 28 days decreased proportionally with the addition of Zn concentration, e.g., 1RM, 2RM, and 3RM. A significant decrease in compressive strength down to −15 MPa was observed compared to the 0RM reference matrix, which indicates negative effects on the dissolution of the cement grains and, consequently, on the hydration coefficient [36,37]. A decrease in the cement amount in the mortars can also influence the sample’s mechanical properties; however, this factor remains negligible compared to the influence of zinc on the hydration of cement.

### 3.4. Heat Hydration Evolution

**Figure 7:**
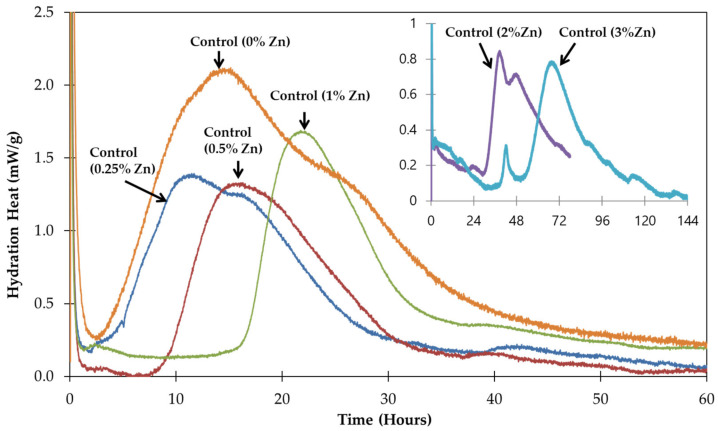
Heat of hydration of different cement pastes doped by 0% to 3% of Zn.

The heat of hydration of the pastes with different Zn substations is presented in Figure 7. The calorimetric variation provides information about the reaction kinetics according to the evolution of Zn concentration. First, when observing the control sample (with 0% Zn) through the lens of conventional hydration stages, the heat calorimetry measured indicates a correlation with the early stages of clinker hydration, specifically C_3_S and C_3_A hydration characterized by exothermal reactions up to 2 h (stage 3) and 12 h (stage 4). Second, specifically for the Zn^2+^ concentration that varied between 0.25% and 1%, the heat of hydration decreased, and the samples passed through stages 3 and 4 more and more rapidly compared to the control sample (0% of Zn). At this concentration interval, the effect of Zn is clearly observed in the degree of hydration of C_3_S and C_3_A and as a delay in the start of the reaction. The initial hydration of C_3_S required 5 h for 025RM, 10 h for 05RM, and 18 h for 1RM. The third interval is partially characterized by a complex perturbation in the hydration of paste, which produces an immediate exothermal reaction that decreases after the hydration of C_3_S and C_3_A is produced. This interval is also characterized by the hydration of C_3_A and C_3_S at 24 and 40 h for 2RM and 3RM, respectively. The compressive strength at 24 h shows a correlation with the hydration results (Figure 3). The reduced compressive strength and the degree of absence of heat at the hydration concentration of Zn are clearly noted.

### 3.5. Mineralogical Properties

**Figure 8:**
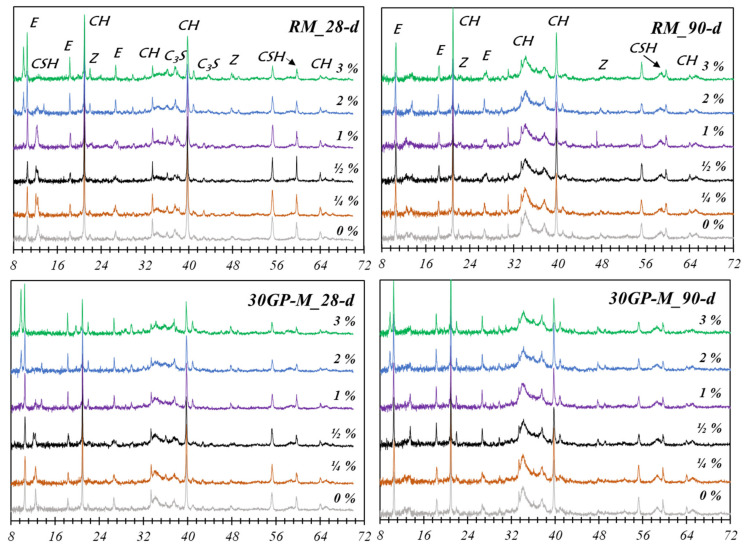
XRD analyses (2θ°) in different Zn concentrations for the RM and 30GP-M, where E = Ettringite and Z = CaZn_2_(OH)_6_·2H_2_O.

XRD analyses were performed at 28 and 90 days, as represented in Figure 8. The results show the occasional appearance of certain precipitation phases of Zn(OH)_2_, CaZn_2_(OH)_6_·2H_2_O and Ca(OH)_2_, hydrozincate CaZn_2_(OH)_6_·2H_2_O, and hydrozincite Zn_5_(OH)_6_(CO_3_)_2_. Zn^2+^ takes several forms depending on its chemical ties with its environment. The presence of C_3_A also promotes the formation of hydrozincate-dehydrated (CaZn_2_(OH)_6_·2H_2_O) [37,38].

The hydraulic binders are characterized by a basic medium rich in Ca^2+^ and the presence of zinc-form compounds of γ-Zn(OH)_2_ and Zn-Ca type. Curing Zn-Si bonds, such as C–S–H gel (in the interlayer), form a simple sorption as no exchange has been observed with Ca^2+^ [39]. This was confirmed by Moudilou [23], explained by trapping in the C–S–H gel interlayers with a fixing mechanism on the outer silicon of the C–S–H gel. The presence of amorphous silicon in the glass powder GP leads to a stable chemical bond with zinc (SiOZn), which explains the appearance of crystallization peaks in the samples with high Zn content. Moulin et al. [28] show that Zn stabilizes on tetrahedral silicon SiOZn. Amorphous silica in solid solutions promotes Zn-Si(GV)-type bonds (in the amorphous silicate in GP).

The amphoteric characteristic of Zn allows it to form morphological variations depending on the pH and the concentration of Zn in the solution. For a very high pH corresponding to that of cement (~13), Zn takes the form Zn(OH)_2_; for a pH less than 13.65, Zn takes the form of ${Zn(OH)}_{4}^{2-}$ [23]. The ionic form of Zn (Zn^2+^) exhibits strong reactivity, translated as amorphous phases formed at the aggregate surface. These phases are characterized by a waterproof surface that delays the hydration binder [36,37,40,41].

- 13.65 < pH

$${Zn(OH)}_{4}^{2-}+{4H}_{3}O^{+}+{2e}^{-}\to Zn+{8H}_{2}O$$

$$E=E_{0}\;{Zn(OH)}_{4}^{2-}/Zn+{0.03\log\left[Zn\left.{\left(OH\right)}_{4}^{2-}\right]\right.\left[H_{3}\right.\left.O^{+}\right]}_{4}$$

E=E0     Zn(OH)42−/Zn−0.12 pH+0.03 log⁡Zn(OH)42−

E=E0     Zn(OH)42−/Zn−0.12 pH+0.03 log⁡0.02

- 6.55 < pH < 13.65

$${Zn(OH)}_{2}+{2H}_{3}O^{+}+{2e}^{-}\to Zn+{4H}_{2}O$$

E=E0     Zn(OH)2Zn+0.03 log⁡H3O+2

$$E=E_{0}\;\frac{{Zn(OH)}_{2}}{Zn}-0.06\;pH$$

In basic solutions, Zn(OH)_2_ takes several crystalline forms. For a pH less than 12, Zn(OH)_2_ has an insoluble amphoteric character. For a pH of 12.8, some researchers suggest that Zn exhibits a contradictory effect because both Zn hydroxides are only formed in the presence of OH^−^. In other words, after the hydration of the main phases of C_3_S and C_2_S cement in acidic solutions, the presence of Zn slows the hydration of these cementitious phases [42]. However, the rapid precipitation of Zn(OH)_2_ on the anhydrous grains, which occurs within a few hours, creates setting delays that can continue for several days. The hydration of the anhydrous phases, therefore, depends on the permeability of this film and on the pH, or the initial formation of Ca^2+^. Incidentally, OH^−^ is dislocated during the transformation of Zn(OH)_2_ into CaZn_2_(OH)_2_·2H_2_O and reinforces the encapsulation of the C_3_S grains, preventing the transport of water, which is necessary for their continuous hydration [43]. The first has been the calcium-crystallized former [42].

2Zn(OH)_3−_ + Ca^2+^ + 2H_2_O → Ca Zn_2_ (OH)_6_, 2H_2_O

Intermediate appearances of Zn(OH)_2_

Zn^2+^ + 2HO− ↔ Zn(OH)_2_ ↔ ZnO(s) + H_2_O (6)

Ks = [Zn^2+^][OH^−^]^2^ (7)

### 3.6. TG Analyses on Hardened Pastes

**Figure 9:**
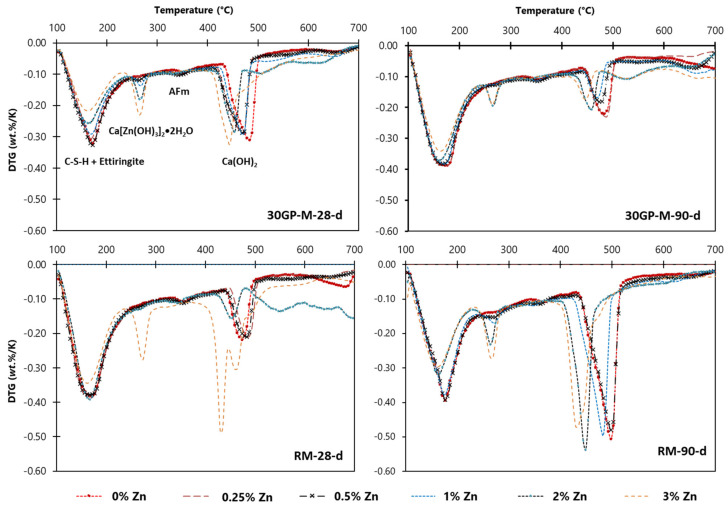
DTG cures of cement pates (100–700 °C).

TG analyses for samples cured between 28 and 90 days show that the presence of Zn^2+^ and N_2_O_6_^2−^ in different concentrations influences the degree of hydration of the cement phases (Figure 9). The results show a change in the loss of mass over temperature intervals in relation to the concentration of Zn. For the two curing times, the matrices develop Zn–AFm compounds (characterized by mass losses between 250 °C and 300 °C) as a function of the Zn concentration. The formation of other hydrates decreases with the Zn concentration increase, such as the formation of C–S–H gel hydration gels (mass losses at 150 °C) and Portlandite CH (mass losses between 450 °C and 600 °C). This observation remains compatible with the blocking nature of the dissolution of C_3_S. The increased amount of Zn led to small decreases in decomposition temperature at the hydrate compounds. The evolution of samples containing 0% to 3% Zn involved a decrease in temperature at 50 °C and 25 °C for the degradation of compounds presented at 50 °C and 180 °C, respectively.

### 3.7. FTIR Analyses

**Figure 10:**
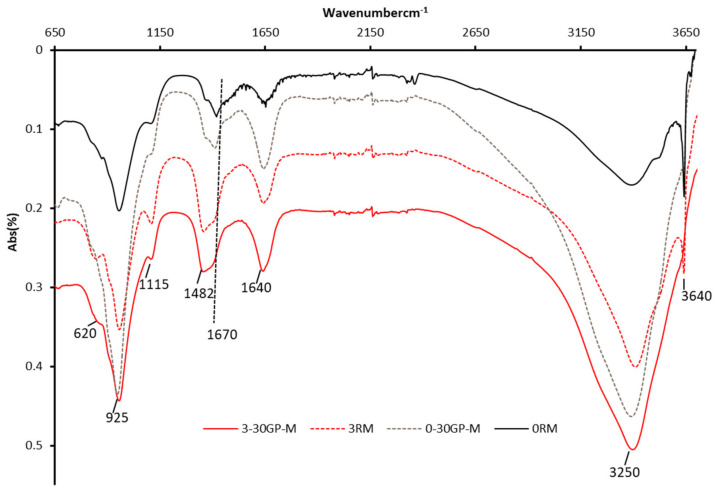
IR patterns (spectra) of cement paste without and with 3% of Zn.

The presence of Zn in the hydrate products of cement leads to the appearance of bands specific to zincate compounds. In this part, the objective is to identify the stable compounds (samples cured for 90 days). Hence, four samples were analyzed, namely the hydrated pastes without Zn (100% CEM I 52.5 N and 70% CEM I 52.5 N + 30GP) and with 3% of Zn. The results are presented in Figure 10. FTIR analyses of Zn-based compounds, such as calcium zincate Ca[Zn(OH)_3_]_2_·2H_2_O, show identical results or IR curves for the hydrated cement pastes (95% clinker), such as those shown in Figure 10, with wavenumbers between 3000 and 3600 cm^−1^, which are related to the hydration cement products [44]. The spectra characterized by peaks at 650 and 1650 cm^−1^ are shared by the hydration products and non-hydrated cement phases [28,41,45]. The 620 and 1670 cm^−1^ wavenumber boundaries are specific to the combination of hydrated cement phases and Zn.

### 3.8. SEM–EDS Analyses

SEM analysis was conducted to observe the GP–paste interface for sample 30GP-M at 3% Zn. As shown in Figure 9, several types of reactions were observed. The first type was in the GP particles, where nucleation sites were observed on the glass surface. The horizontal observation shows two types of morphological germination: spherical and tubular-shaped. These shapes are not related to the initial shape of the glass particles, which results in the germination of the GP. The vertical observation shows inter-diffusion between the GP and the chemical composition of the exterior phases. Beyond PG, more than the classic form of hydration of cement, the filleted structures. The presence of Ca^2+^ and OH- in high concentrations in the cement leads to the formation of a stable phase of calcium hydroxyzincate as a precipitate, which allows the Zn to pass from the amorphous structure (Zn(OH)_2_) to the calcium hydroxyzincate-type crystalline structure, according to the reaction below:

2 Zn(OH)^2−^ +Ca^2+^ + 2 H_2_O·CaZn(OH)_4_, 2 H_2_O (8)

Calcium zincate can take several forms, depending on the treatment conditions and the concentration of elements, with shapes varying from haliatory leaves to well-structured leaves [46].

**Figure 11:**
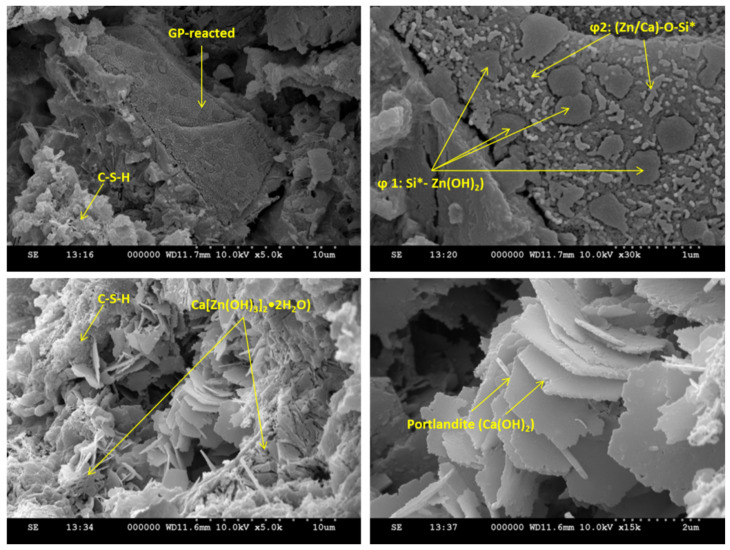
SEM images of sample 3MR3PG were cured for 90 days.

**Figure 12:**
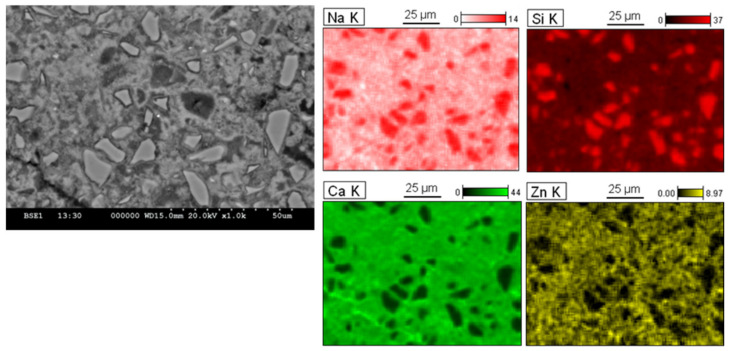
SEM–EDS analysis of polished surface sample 3MR3PG cured for 90 days.

The SEM–EDS analyses were conducted on the 30GP-M-90d paste (Figure 11 and Figure 12). These samples show rich Ca and Zn germination around GPs. The gray spots indicate anhydrate cement particles that are surrounded by Zn. Around the GP particles, the compatibility of germination between Zn and Ca around GPs. The localization of Zn in the hydrated paste is almost homogeneous, which was explained by the addition of mixing water when the hydration had started. Finally, sorption isotherms of Zn^2+^ were conducted on unhydrated (C_3_S), hydrated gel (C–S–H gel), and on the GP surface (Si*–O–Zn). Two types of Zn germination are observed on the surfaces of the GP, noted as *ϕ*_1_ and *ϕ*_2_. Si*-(ZN(OH)_2_) was observed to be bulky compared to the compounds of the Zn–O–Si type [47].

## 4. Discussion

**Figure 13:**
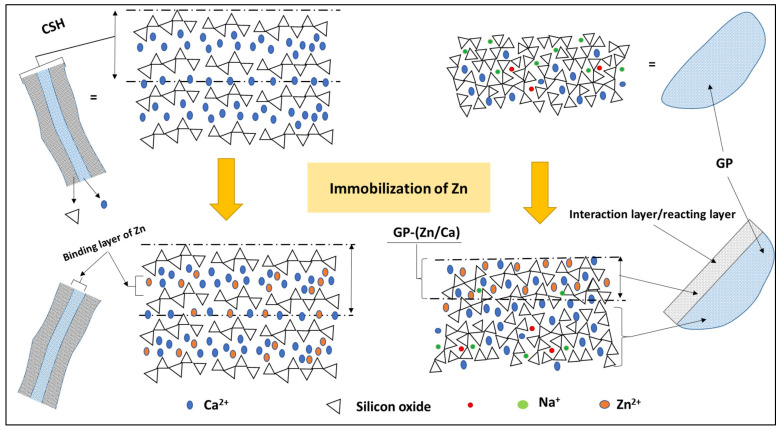
Approximate model of Zn^2+^ fixation on C–S–H gel and glass aggregates.

An approximate model of the binding of Zn^2+^ by the C–S–H gel hydration phase resulting from the hydration of the cement as well as by the glass aggregates is given in Figure 13. Microstructure studies and approaches described in the literature [28,48] make it possible to propose methods of binding Zn in the structure of C–S–H gel. Note that the glass aggregates after 90 days of cure are also involved in the fixing of Zn. The presence of GP gives a new mode of fixation of Zn^2+^, and the GP surface can immobilize this metal cation and help the hydration of cement to start progressively. The presence of Zn^2+^ in a cementitious matrix leads to the formation of an impermeable film around the cement and glass particles, which blocks their hydration and subsequently hinders the process of forming a hydration gel (C–S–H) in large quantities. This impermeabilization primarily occurs at the interface, where Zn integrates into the C–S–H gel layers. In the presence of Ca^2+^, glass undergoes pozzolanic reactivity, while the presence of Zn^2+^ introduces competition on the reaction surface with glass. This results in a strong dynamism in the reaction mechanism and the amount of Zn that is fixed. This mechanism is not well documented in the literature. Based on analytical results and the reaction mechanism of GP on the contact surface, Figure 13 presents an approximate mechanism of GP reactivity as well as cement grain interactions.

## 5. Conclusions

This study shows the advantage of using glass in combination with hydraulic binders for Zn stabilization. The results of this study are detailed below.

- The presence of Zn in hydroxide form influences the setting time of Portland cement. Delays in the setting time can occur up to 24 h (for Zn concentrations between 2% and 3% relative to the cement). The presence of glass only influences the setting properties after ~90 days of curing. For shorter hardening times, the reactivity of the glass is lower than that of the cement particles. The high concentration of Zn (from 2%) significantly retarded the hydration of C_3_S (>40 h).
- The glass aggregates have the capacity to immobilize Zn after 90 days of curing. The glass forms irreversible phases on the layers in contact with the pastes. Stabilization only occurs through inter-diffusion or dissolution in a solid base of GP. TG analysis shows the influence of GP on the formation of hydrated phases, which is crucial for Zn remediation. As such, the presence of glass promotes hydration reactions. SEM–EDS analyses confirmed surface germination reactions, and the chemical analyses confirmed the presence of phases rich in both Zn and Ca.
- The microstructural studies and approaches described in the literature [28,48] make it possible to propose methods of binding Zn in the structure of C–S–H gel. Note that glass aggregates after 90 days of curing are also involved in the fixing of Zn.
- The presence of GP provides a new pathway for the fixation of Zn^2+^. The GP surface can immobilize the metal cation and help the hydration of cement to start gradually.

## Figures and Tables

**Table 1:** Physicochemical properties of the used powders.

Parameters		GP	CEM	Standard/Method
Physical Properties	Absolute density (g/cm^3^)	2.54	3.15	NF EN 1097-7 [31]
	SSA BET (m^2^/kg)	793	835	NF EN ISO18757 [32]
	Fire loss % (450 °C/3 h)	0.02	–	NF EN ISO 17892-12 [33]
	Fire loss % (550 °C/1 h)	0.03	–	NF EN 15169 [34]
	Fire loss % (1000 °C/1 h)	–	0.30	NF EN 1097-7 [31]
Chemical Composition wt.%	SiO_2_	70.8	17.4	measured by XRF
	Al_2_O_3_	1.70	4.44	
	MgO	1.20	0.85	
	Fe_2_O_3_	0.40	2.97	
	CaO	11.5	60.7	
	Na_2_O	13.0	0.31	
	K_2_O	0.70	1.16	
	SO_3_	–	3.75	
	Others	0.01	0.90	

**Table 2:** Details of the Bangham fit (solid lines) are reported in Figure 5.

	Zn/Binder w%	Y = Log(Qt)	Log(kd)	kd	R^2^
Reference Mortar	0.25%	y = −0.46x − 2.82	−2.82	1.50 × 10^−3^	0.98
	0.50%	y = −0.46x − 2.44	−2.44	3.64 × 10^−3^	0.98
	1%	y= −0.36x − 2.24	−2.24	5.75 × 10^−3^	0.88
	2%	y = −0.40x − 1.57	−1.57	2.70 × 10^−2^	0.97
	3%	y = −0.49x − 1.29	−1.29	5.13 × 10^−2^	0.99
Blended Mortar	0.25%	y = −0.64x − 2.34	−2.34	4.53 × 10^−3^	0.98
	0.50%	y = −0.50x − 2.07	−2.07	8.47 × 10^−3^	0.78
	1%	y = −0.51x − 1.81	−1.81	1.55 × 10^−2^	0.98
	2%	y = −0.60x − 1.22	−1.22	6.00 × 10^−2^	0.99
	3%	y = −0.60x − 1.00	−1.00	9.88 × 10^−2^	0.92

## Data Availability

The original contributions presented in the study are included in the article, further inquiries can be directed to the corresponding author.

## References

[1] Bouchikhi A., Benzerzour M., Abriak N.E., Maherzi W., Mamindy-Pajany Y. (2019). Study of the Impact of Waste Glasses Types on Pozzolanic Activity of Cementitious Matrix. Constr. Build. Mater..

[2] Idir R., Cyr M., Tagnit-Hamou A. (2010). Use of Fine Glass as ASR Inhibitor in Glass Aggregate Mortars. Constr. Build. Mater..

[3] Lee H., Hanif A., Usman M., Sim J., Oh H. (2018). Performance Evaluation of Concrete Incorporating Glass Powder and Glass Sludge Wastes as Supplementary Cementing Material. J. Clean. Prod..

[4] Ling T.C., Poon C.S., Wong H.W. (2013). Management and Recycling of Waste Glass in Concrete Products: Current Situations in Hong Kong. Resour. Conserv. Recycl..

[5] (2020). Standard Specification for Ground-Glass Pozzolan for Use in Concrete.

[6] Zidol A. (2014). Durabilité En Milieux Agressifs des Bétons Incorporant la Poudre de Verre.

[7] Dali J.S., Tande S.N. Performance of Concrete Containing Mineral Admixtures Subjected to High Temperature. Proceedings of the 37th Conference on Our World in Concrete and Structures.

[8] Bouchikhi A., Mamindy-Pajany Y., Maherzi W., Albert-Mercier C., El-Moueden H., Benzerzour M., Peys A., Abriak N. (2020). Use of Residual Waste Glass in an Alkali-Activated Binder—Structural Characterization, Environmental Leaching Behavior and Comparison of Reactivity. J. Build. Eng..

[9] Bouchikhi A., Maherzi W., Benzerzour M., Mamindy-pajany Y., Peys A. (2021). Manufacturing of Low-Carbon Binders Using Waste Glass and Dredged Sediments: Formulation and Performance Assessment at Laboratory Scale. Sustainability.

[10] Torres-Carrasco M., Puertas F. (2015). Waste Glass in the Geopolymer Preparation. Mechanical and Microstructural Characterisation. J. Clean. Prod..

[11] Luhar S., Cheng T.W., Nicolaides D., Luhar I., Panias D., Sakkas K. (2019). Valorisation of Glass Wastes for the Development of Geopolymer Composites—Durability, Thermal and Microstructural Properties: A Review. Constr. Build. Mater..

[12] El-Kamash A.M. (2008). Evaluation of Zeolite A for the Sorptive Removal of Cs+ and Sr2+ Ions from Aqueous Solutions Using Batch and Fixed Bed Column Operations. J. Hazard. Mater..

[13] Osmanlioglu A.E. (2006). Treatment of Radioactive Liquid Waste by Sorption on Natural Zeolite in Turkey. J. Hazard. Mater..

[14] Davis M.E. (1991). Zeolites and Molecular Sieves: Not Just Ordinary Catalysts. Ind. Eng. Chem. Res..

[15] Katzer J.R. (1989). Atlas of Zeolite Structure Types. W. M. Meier, and D. H. Olson, 2nd Rev. Ed., Butterworth, 1987. AIChE J..

[16] Nikolići I., Dsignurović D., Tadić M., Blečić D., Radmilović V. (2013). Immobilization of Zinc from Metallurgical Waste and Water Solutions Using Geopolymerization Technology. E3S Web Conf..

[17] Ziegler F., Johnson C.A. (2001). The Solubility of Calcium Zincate (CaZn_2_(OH)_6_·2H_2_O). Cem. Concr. Res..

[18] Deschamps T., Benzaazoua M., Bussière B., Belem T., Mbonimpa M. (2006). Mécanismes de Rétention Des Métaux Lourds En Phase Solide: Cas de La Stabilisation Des Sols Contaminés et Des Déchets Industriels. VertigO.

[19] Shi C., Fernández-Jiménez A. (2006). Stabilization/Solidification of Hazardous and Radioactive Wastes with Alkali-Activated Cements. J. Hazard. Mater..

[20] Arliguie G., Grandet J. (1990). Influence de la Composition D’un Ciment Portland Sur Son Hydratation en Presence de Zinc. Cem. Concr. Res..

[21] Hills C.D., Pollard S.J.T. (1997). The Influence of Interference Effects on the Mechanical, Microstructural and Fixation Characteristics of Cement-Solidified Hazardous Waste Forms. J. Hazard. Mater..

[22] El-Eswed B.I. (2016). Solidification Versus Adsorption for Immobilization of Pollutants in Geopolymeric Materials: A Review. Solidification.

[23] Moudilou E. (2003). Cinetiques et Mecanismes de Relargage des Metaux Lourds Presents en Traces dans les Matrices Cimentaires. Ph.D. Thesis.

[24] Gineys N., Aouad G., Damidot D. (2010). Managing Trace Elements in Portland Cement—Part I: Interactions between Cement Paste and Heavy Metals Added during Mixing as Soluble Salts. Cem. Concr. Compos..

[25] Chen Q.Y., Tyrer M., Hills C.D., Yang X.M., Carey P. (2009). Immobilisation of Heavy Metal in Cement-Based Solidification/Stabilisation: A Review. Waste Manag..

[26] Abdulhussein Saeed K., Anuar Kassim K., Eisazadeh A. (2012). Interferences of Cement Based-Solidification/Stabilization and Heavy Metals: A Review. Electron. J. Geotech. Eng..

[27] Panfili F. (2016). Etude de l’évolution de La Spéciation Du Zinc Dans La Phase Solide d’un Sédiment de Curage Contamine. Ph.D. Thesis.

[28] Moulin I., Stone W.E.E., Sanz J., Bottero J.Y., Mosnier F., Haehnel C. (1999). Lead and Zinc Retention during Hydration of Tri-Calcium Silicate: A Study by Sorption Isotherms and 29Si Nuclear Magnetic Resonance Spectroscopy. Langmuir.

[29] (2020). Particle Size Analysis—Laser Diffraction Methods.

[30] (2016). Methods of Testing Cement—Part 1: Determination of Strength.

[31] (2022). Tests for Mechanical and Physical Properties of Aggregates—Part 7: Determination of the Particle Density of Filler—Pyknometer Method.

[32] (2006). Fine Ceramics (Advanced Ceramics, Advanced Technical Ceramics)—Determination of Specific Surface Area of Ceramic Powders by Gas Adsorption Using the BET Method.

[33] (2018). Geotechnical Investigation and Testing—Laboratory Testing of Soil—Part 12: Determination of Liquid and Plastic Limits.

[34] (2004). Petroleum and Liquid Petroleum Products—Determination of Volume, Density and Mass of the Hydrocarbon Content of Vertical Cylindrical Tanks by Hybrid Tank Measurement Systems.

[35] Snellings R., Cizer Ö., Horckmans L., Durdzi T., Dierckx P., Nielsen P., Van Balen K., Vandewalle L. (2016). Applied Clay Science Properties and Pozzolanic Reactivity of Fl Ash Calcined Dredging Sediments. Appl. Clay Sci..

[36] Fernández Olmo I., Chacon E., Irabien A. (2001). Influence of Lead, Zinc, Iron (III) and Chromium (III) Oxides on the Setting Time and Strength Development of Portland Cement. Cem. Concr. Res..

[37] Deschamps T. (2009). Étude Du Comportement Physique Et Hydrogéochimique D’Un Dépôt De Résidus Miniers En Pâte Dans Des Conditions De Surface. Ph.D. Thesis.

[38] Brault S. (2001). Modélisation Du Comportement à La Lixiviation à Long Terme de Déchets Stabilisés à l’aide de Liants Hydrauliques. Ph.D. Thesis.

[39] Ziegler F., Scheidegger A.M., Johnson C.A., Dáhn R., Wieland E. (2001). Sorption Mechanisms of Zinc to Calcium Silicate Hydrate: X-Ray Absorption Fine Structure (XAFS) Investigation. Environ. Sci. Technol..

[40] Kakali G., Tsivilis S., Tsialtas A. (1998). Hydration of Ordinary Portland Cements Made from Raw Mix Containing Transition Element Oxides. Cem. Concr. Res..

[41] McWhinney H., Cocke D.L., Yu G.S. (1989). Solidification of Hazardous Substances-a Tga and Ftir Study of Portland Cement Containing Metal Nitrates. J. Environ. Sci. Health Part A Environ. Sci. Eng..

[42] Arliguie G., Ollivier J.P., Grandet J. (1982). Etude de l’effet Retardateur Du Zinc Sur l’hydratation de La Pate de Ciment Portland. Cem. Concr. Res..

[43] Šiler P., Kolárová I., Másilko J., Novotný R., Opravil T. (2018). The Effect of Zinc on the Portlands Cement Hydration. Key Eng. Mater..

[44] Li S., Zhou Y. (2012). Preparation of Tetragonal and Hexagonal Calcium Zincate. Appl. Mech. Mater..

[45] Farcas F., Touzé P. (2001). La Spectrométrie Infrarouge à Transformée de Fourier (IRTF). Bull. Lab. Ponts Chaussées.

[46] Shangguan E., Li L., Wu C., Fu P., Wang M., Li L., Zhao L., Wang G., Li Q., Li J. (2021). Microemulsion Synthesis of 3D Flower-like Calcium Zincate Anode Materials with Superior High-Rate and Cycling Property for Advanced Zinc-Based Batteries. J. Alloys Compd..

[47] Reddy V.A., Solanki C.H., Kumar S., Reddy K.R., Du Y.J. (2019). New Ternary Blend Limestone Calcined Clay Cement for Solidification/Stabilization of Zinc Contaminated Soil. Chemosphere.

[48] Yang Y., Zhao T., Jiao H., Wang Y., Li H. (2020). Potential Effect of Porosity Evolution of Cemented Paste Backfill on Selective Solidification of Heavy Metal Ions. Int. J. Environ. Res. Public Health.

